# Dr. Warren’s Address before the American Medical Association

**Published:** 1850-11

**Authors:** 


*Dr. Warren's Address before the American Medical Association.*—The Address delivered by Dr. Warren at Cincinnati, at the opening of the session of the American Medical Association, has been published by John Wilson, of Boston, Mass., in beautiful style. The Address was delivered *memoriter*, and not furnished for publication with the Transactions—an omission to be regretted. The printed Address contains much that was not spoken, and, altogether, it does the author better justice in its present form, than as delivered *viva voce.*

